# Identification, Geographical Traceability, and Thermal Oxidation and Photodegradation Studies of Camellia Oil Based on Raman Spectroscopy

**DOI:** 10.3390/molecules30112473

**Published:** 2025-06-05

**Authors:** Boxue Chang, Jingyue Huang, Qingli Xie, Yinlan Ruan, Rukuan Liu

**Affiliations:** 1 School of Electronic Engineering and Automation, Guilin University of Electronic Technology, Guilin 541004, China; 409962832@guat.edu.cn (B.C.); huangjy0230@163.com (J.H.); 2 Engineering Comprehensive Training Center, Guilin University of Aerospace Technology, Guilin 541004, China; 3 School of Optoelectronic Engineering, Guilin University of Electronic Technology, Guilin 541004, China; xieql8009@163.com; 4 Guangxi Key Laboratory of Optoelectronic Information Processing, Guilin 541004, China; 5 State Key Laboratory of Utilization of Woody Oil Resource, Hunan Academy of Forestry, Changsha 410004, China

**Keywords:** camellia oil, Raman spectroscopy, Partial Least Squares Discriminant Analysis (PLS-DA), Multivariate Curve Resolution-Alternating Least Squares (MCR-ALS), geographical traceability, thermal oxidation, photodegradation

## Abstract

Camellia oil, rich in monounsaturated fatty acids, squalene, tocopherols, and polyphenols, is highly valued for its nutritional benefits. However, its high market value and regional variations have led to frequent adulteration, highlighting the need for rapid, non-destructive methods for authentication, geographical traceability, and quality assessment. This study employed portable Raman spectroscopy combined with Partial Least Squares Discriminant Analysis (PLS-DA) and Multivariate Curve Resolution–Alternating Least Squares (MCR-ALS) to differentiate camellia oil from other edible oils and evaluate its thermal and photo-oxidative stability. PLS-DA, based on VIP-selected spectral variables, effectively distinguished camellia oil, with Raman bands near 1250 cm^−1^ and 1650 cm^−1^ contributing significantly. A unique peak at 1525 cm^−1^, observed in samples from Gongcheng, Guangxi, was associated with carotenoids and served as a potential marker for geographical traceability. MCR-ALS modeling revealed significant reductions in the 1650 cm^−1^ and 1525 cm^−1^ peaks when temperatures exceeded 150 °C, indicating degradation of unsaturated fatty acids and carotenoids. Under UV exposure, the 1525 cm^−1^ peak declined sharply and nearly disappeared after 24 h, suggesting rapid carotenoid degradation via photooxidation. Extended UV treatment also affected the 1650 cm^−1^ peak and led to oxidative product accumulation. Overall, this study demonstrates the feasibility of integrating Raman spectroscopy with chemometric analysis for efficient oil classification, traceability, and stability monitoring, offering a valuable tool for food quality control and market supervision.

## 1. Introduction

Camellia oil, extracted mainly from the seeds of Camellia oleifera, is a high-value edible oil rich in monounsaturated fatty acids (MUFA), squalene, vitamin E, and polyphenols [1,2]. It is primarily produced in southern China, especially in the Guangxi, Jiangxi, and Hunan provinces. According to Liang et al. [3], the annual output of Camellia oleifera fruits in China exceeds 2.4 million tons, reflecting its substantial production scale and economic importance. Due to its high oleic acid content (>80%) and antioxidant components, camellia oil has earned the nickname “eastern olive oil” and is recognized for its potential health benefits, including cardiovascular protection and anti-inflammatory effects [4,5]. However, its high market value and limited supply make it vulnerable to adulteration. Moreover, camellia oil is susceptible to degradation under high-temperature processing and light exposure, which may affect its chemical composition and quality stability, compromising its nutritional quality. Therefore, reliable techniques for authentication, geographical traceability, and stability assessment are crucial for quality control and food safety assurance.

High-temperature cooking methods, such as frying, boiling, microwaving, and baking, are inevitable steps in oil utilization. However, under high-temperature conditions, oils undergo hydrolysis, oxidation, and polymerization, which alter their sensory properties and nutritional value [6,7,8,9]. Oxidation, in particular, is the primary factor causing oil quality deterioration, leading to the formation of various oxidation products, such as hydroperoxides, aldehydes, ketones, hydrocarbons, and alcohols [10,11]. These oxidation products not only affect the flavor of the oil but may also pose potential health risks. The oxidation stability of camellia oil is primarily influenced by its chemical composition, especially the type and content of unsaturated fatty acids. The higher the degree of unsaturation, the more prone the oil is to oxidative degradation [12,13]. Thus, systematically investigating the thermal oxidation behavior of camellia oil and its stability is crucial for optimizing its culinary applications and quality control.

Apart from thermal processing, light exposure is another critical factor affecting camellia oil quality. Ultraviolet (UV) and visible light exposure can trigger lipid photooxidation, leading to the degradation of unsaturated fatty acids and carotenoids, thereby reducing the oil’s antioxidant capacity and storage stability [14,15]. Carotenoids are essential antioxidants in camellia oil, and their photodegradation can significantly impact its oxidative stability [16]. Moreover, light-induced oxidation may accelerate the accumulation of oxidation products, further compromising oil quality. However, current research on the photodegradation behavior of camellia oil remains limited, necessitating further exploration of its degradation mechanisms and impact on quality.

The increasing market demand has also posed another major challenge for camellia oil—adulteration. Due to its high price, camellia oil is frequently adulterated with low-cost edible oils, such as rapeseed oil, palm oil, and corn oil, undermining consumer rights and diminishing the health benefits of genuine camellia oil [17,18]. Additionally, the chemical composition of camellia oil is influenced by geographical origin, plant varieties, and processing methods. Oils from different regions exhibit variations in fatty acid composition, secondary metabolites, and volatile compounds [19,20]. Hence, establishing reliable authentication and geographical traceability techniques is essential for maintaining market integrity and ensuring product quality.

Traditional edible oil analysis methods primarily rely on gas chromatography–mass spectrometry (GC-MS) [21,22,23] and high-performance liquid chromatography (HPLC) [24,25,26] techniques, which offer high-precision chemical composition analysis. However, these methods require complex sample preparation, lengthy analysis times, and high operational costs, making them impractical for real-time food safety monitoring. In contrast, Raman spectroscopy, a non-destructive, rapid, and highly sensitive molecular spectroscopy technique, provides direct molecular vibration information for sample identification [27,28] and structural analysis [29,30,31]. In recent years, Raman spectroscopy has shown immense potential in edible oil quality control applications [32,33,34].

**Figure 1:**
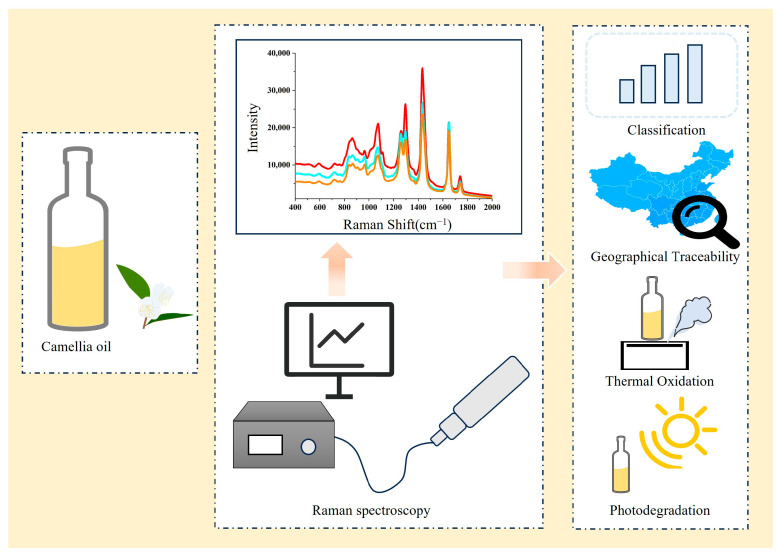
Graphical summary of Raman spectroscopy-based classification, traceability, and stability analysis of camellia oil.

This study utilizes Raman spectroscopy coupled with multivariate statistical analysis methods, including PLS-DA and MCR-ALS, to systematically explore the relationships among authentication, geographical origin, and quality stability of camellia oil (Figure 1). These three aspects—identification, traceability, and oxidative stability—are intrinsically linked, as the compositional differences driven by geographical factors not only affect the Raman spectral fingerprint for authentication but also influence the oil’s resistance to thermal and photodegradation. Specifically, Raman spectral features were first employed to classify camellia oil against common edible oils based on inherent compositional differences. Subsequently, the correlation between unique Raman spectral markers and geographical origin was investigated to establish robust traceability. Finally, thermal oxidation and photodegradation behaviors were examined to reveal how compositional characteristics influenced by geographical factors impact the oil’s chemical stability. By integrating these analytical aspects, this research provides comprehensive insights into the intrinsic links between origin-related composition, authenticity, and stability, supporting more effective quality control and food safety management strategies for camellia oil.

## 2. Results and Discussion

### 2.1. Raman Spectral Characteristics of Camellia Oil

**Figure 2:**
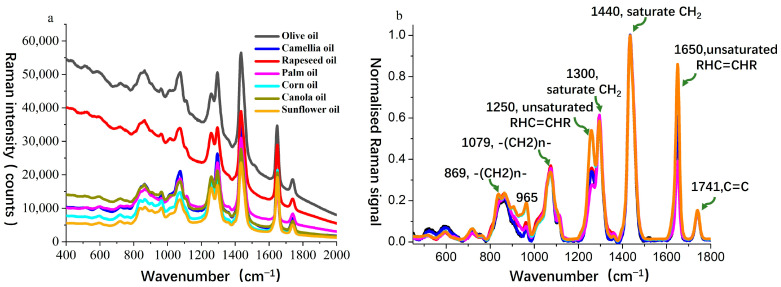
Raman spectra of oil samples: (**a**) raw Raman spectra of seven types of edible oils; (**b**) normalized Raman spectra highlighting comparative spectral features.

For Sample Set 1, Raman spectra were obtained for seven types of oils, including camellia oil, olive oil, and vegetable oils (rapeseed oil, palm oil, corn oil, canola oil, and sunflower oil), as shown in Figure 2a. The spectral range of 700–1800 cm^−1^ was selected for analysis as this region is commonly referred to as the fingerprint spectral region, where significant Raman features occur for edible oils. As illustrated in Figure 2a, all oil samples exhibited their highest intensity at the 1440 cm^−1^ peak. Therefore, all spectra were normalized to this peak, which corresponds to the scissoring bending vibration mode of the -CH_2_ group [35].

Since different oils are primarily composed of triglycerides and straight-chain fatty acids with 16–18 carbon atoms, their Raman spectral peak positions in the wavenumber range of 960–1800 cm^−1^ are highly similar. According to peak assignment methods in the literature [36,37], the characteristic bands are interpreted as follows: 869 cm^−1^ corresponds to the C–C stretching vibration of methylene chains (-(CH_2_)n-); 965 cm^−1^ is assigned to the C=C bending vibration in trans-RHC=CHR groups; 1079 cm^−1^ corresponds to the C–C stretching vibration of methylene chains (-(CH_2_)n-); 1250 cm^−1^ represents the in-plane δ(C–H) deformation vibration of unconjugated cis double bonds; 1300 cm^−1^ corresponds to the symmetric twisting vibration of methylene (-CH_2_-); 1440 cm^−1^ is associated with the scissoring bending vibration of methylene (-CH_2_-) [δ(CH_2_)]; 1650 cm^−1^ represents the C=C stretching vibration of cis double bonds (RHC=CHR); and 1741 cm^−1^ corresponds to the C=O stretching vibration of ester carbonyl groups (RC=OOR). The spectral bands at 1250 cm^−1^ and 1650 cm^−1^ are directly related to the C=C double bond structure and can be used to evaluate oil unsaturation, which is critical for the qualitative and quantitative analysis of oil composition.

**Figure 3:**
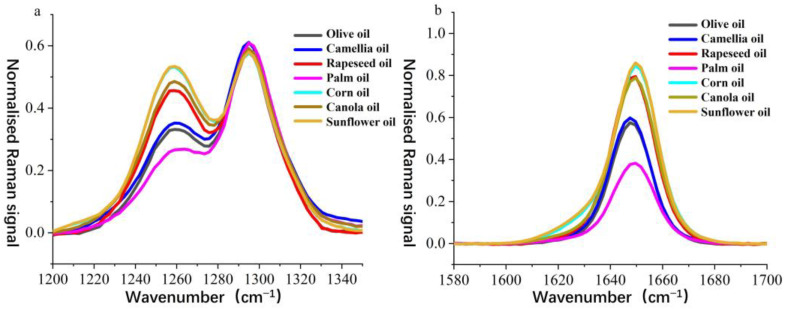
Normalized Raman spectra within the wavenumber range of (**a**) 1200–1340 cm^−1^ and (**b**) 1580–1700 cm^−1^.

To further analyze the spectral differences between camellia oil and other edible oils, the normalized Raman spectra of all samples were compared in detail within the 1200–1340 cm^−1^ (Figure 3a) and 1580–1700 cm^−1^ (Figure 3b) wavenumber ranges. The Raman peaks near 1250 cm^−1^ and 1650 cm^−1^ serve as key distinguishing features among different edible oils, as they are primarily associated with C–C bending vibrations and C=C stretching vibrations in fatty acids [38], providing insight into the molecular structure and composition of the oils.

In the 1250 cm^−1^ wavenumber range (Figure 3a), notable differences in Raman intensity were observed among the edible oils. The signal at this peak for camellia oil was lower than that of rapeseed oil, corn oil, canola oil, and sunflower oil but higher than that of palm oil, showing a similarity to olive oil. This suggests that the C–C vibration modes in camellia oil closely resemble those in olive oil, whereas other vegetable oils exhibited stronger signals, which may be attributed to their higher polyunsaturated fatty acid content. A similar trend was observed in the 1650 cm^−1^ wavenumber range (Figure 3b). This peak is generally attributed to the C=C stretching vibration in unsaturated fatty acids and serves as an indicator of unsaturation levels. The Raman intensity at this peak for camellia oil was again lower than that of rapeseed oil, corn oil, canola oil, and sunflower oil but higher than that of palm oil, remaining closely aligned with olive oil. This indicates that camellia oil has a higher monounsaturated fatty acid ratio than palm oil but a lower polyunsaturated fatty acid content compared to vegetable oils rich in polyunsaturated fatty acids, such as rapeseed oil and sunflower oil.

Based on the comprehensive analysis of the spectral features at 1250 cm^−1^ and 1650 cm^−1^, the Raman signals of camellia oil at these two key bands were found to be intermediate between those of palm oil and vegetable oils rich in polyunsaturated fatty acids while exhibiting a high degree of similarity to olive oil. These spectral differences provided an important basis for the authentication of camellia oil. Combined with PLS-DA modeling, the subtle variations in key Raman peaks enabled the rapid and accurate discrimination of camellia oil from other edible oils, thereby offering strong support for the authenticity verification and quality control of camellia oil.

### 2.2. PLS-DA Classification Model

For the Raman spectral data of Sample Set 1, data preprocessing was first performed, including baseline correction, S-G smoothing, and normalization, to optimize the spectral signals and reduce noise interference. Subsequently, within the spectral range of 700–1800 cm^−1^, significant characteristic bands contributing to classification were selected based on Variable Importance in Projection (VIP) scores (VIP > 1.0), and these selected variables were used as inputs to construct a Partial Least Squares Discriminant Analysis (PLS-DA) model to investigate the spectral differences and classification characteristics among different oil samples.

**Figure 4:**
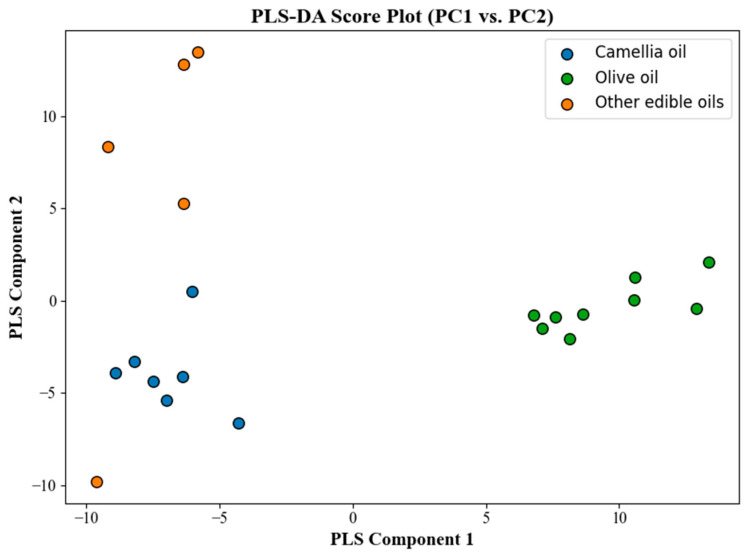
PLS-DA score plot of different oil samples.

Figure 4 shows the PLS-DA score plot, where the sample distribution among different oil categories is clearly observed through the projection of the first two latent variables (LVs).

As shown in the score plot, different oil samples exhibited a good separation trend in the two-dimensional space. The blue samples represent camellia oil from different geographical origins, which are relatively clustered, indicating stable and consistent spectral characteristics within the selected feature bands. The green samples represent other common vegetable oils (such as rapeseed oil, corn oil, and sunflower oil), which are distributed far from the camellia oil samples, reflecting significant differences in chemical composition or molecular structure.

Olive oil (green samples) and camellia oil (blue samples) exhibited a clear separation trend along the LV1 direction, with olive oil samples mainly distributed in the positive region of LV1, while camellia oil samples were concentrated in the negative region. Additionally, differences were also observed along the LV2 direction, further enhancing the classification distinction. These results indicate that although camellia oil and olive oil may show certain similarities in conventional physicochemical indicators, they can be effectively discriminated through Raman spectroscopy combined with PLS-DA modeling. This finding is consistent with the previous literature [39], which reported that while traditional methods struggle to distinguish between camellia oil and olive oil, Raman spectroscopy coupled with multivariate discriminant analysis provides a clearer classification boundary.

**Figure 5:**
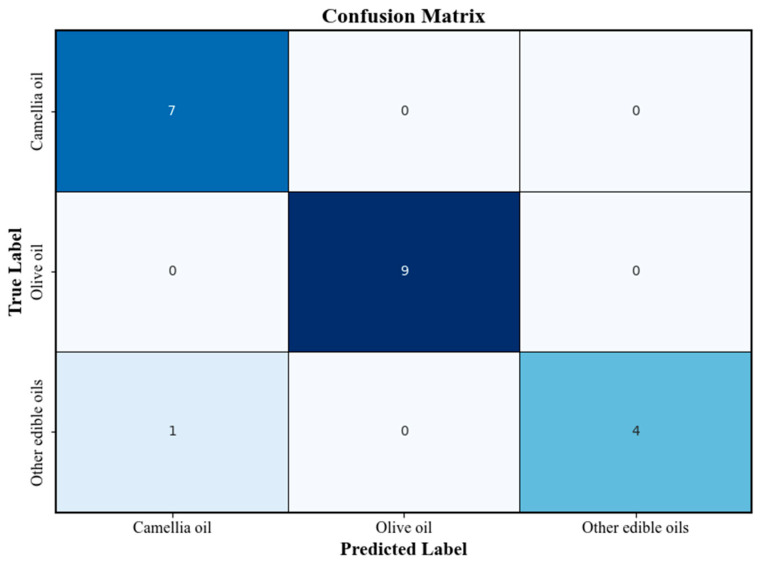
Confusion matrix of the PLS-DA classification model.

Figure 5 presents the confusion matrix of the PLS-DA classification model. The overall classification accuracy exceeded 80%, and high correct recognition rates were achieved for both camellia oil and olive oil samples. The confusion matrix further verified the robustness and reliability of the model in practical sample classification tasks.

### 2.3. Geographical Traceability of Camellia Oil

**Figure 6:**
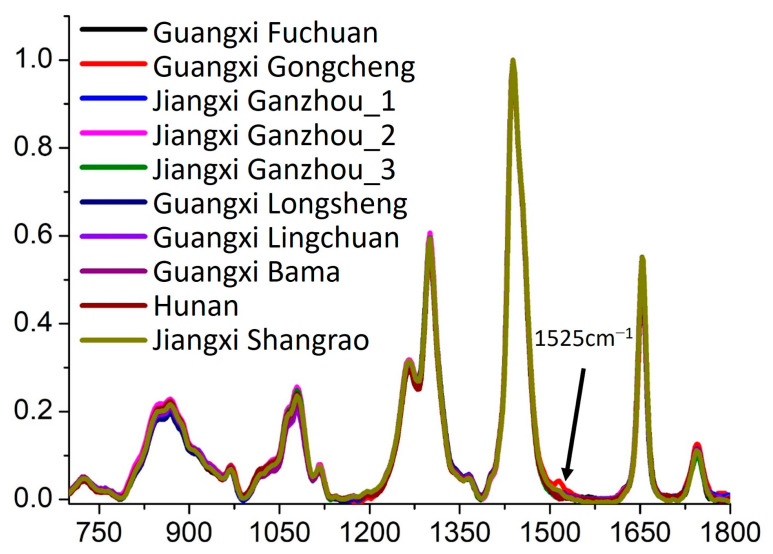
The Raman spectra of the camellia oils from different provinces in China.

To further investigate the spectral characteristics of Sample Set 2, the Raman spectra were analyzed in detail, as shown in Figure 6. The results indicate that, except for the samples from Gongcheng, Guangxi, camellia oil samples from all other regions exhibited highly consistent spectral features characterized by identical Raman peak positions and similar peak intensities. This finding suggests that these samples share high similarity in their primary chemical composition and molecular structure, likely due to similar plant germplasm resources, comparable cultivation environments, or similar processing techniques.

However, camellia oil samples from Gongcheng, Guangxi, exhibited an additional Raman peak at 1525 cm^−1^. This observation indicates a certain degree of chemical composition specificity in these samples. Based on the existing literature and comparisons with the spectral characteristics of olive oil [39,40], we speculate that this Raman peak may be associated with the vibrational mode of carotenoid complexes. Carotenoids, as natural pigments and bioactive compounds, are influenced by multiple factors, including plant variety, growth environment, maturity level, and processing methods. Therefore, the presence of this additional Raman peak may reflect unique characteristics in the cultivation or processing of camellia oil from Gongcheng, Guangxi, such as a higher carotenoid content or specific lipid metabolism pathways. Furthermore, the existence of this characteristic peak provides a potential molecular marker for the geographical traceability of camellia oil. It could be used to identify camellia oil from specific production regions, thereby enhancing market regulation and product quality control.

In summary, Raman spectroscopy analysis effectively differentiates camellia oil from Gongcheng, Guangxi, from those of other regions, demonstrating its significant potential in geographical traceability research. Future studies could integrate high-resolution mass spectrometry (HRMS) and nuclear magnetic resonance (NMR) spectroscopy to further investigate the specific molecular composition of this characteristic peak and its formation mechanism, providing a more comprehensive understanding of geographically specific chemical markers in camellia oil.

### 2.4. Thermal Oxidation and Photodegradation Studies of Camellia Oil

#### 2.4.1. Thermal Oxidation Degradation Study

**Figure 7:**
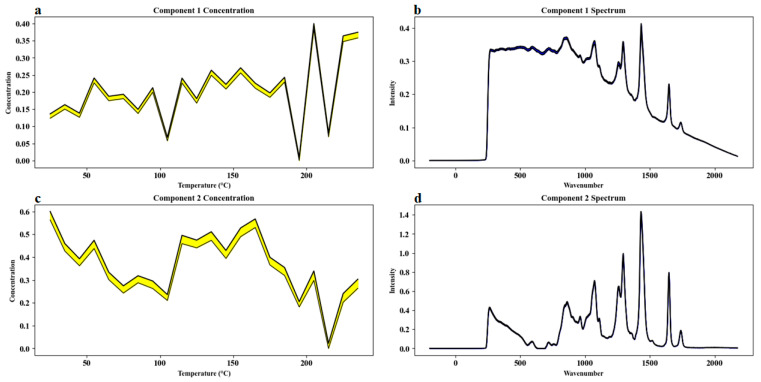
Comprehensive analysis of camellia oil heating process data using Multivariate Curve Resolution (MCR) method: (**a**) concentration profile of Component 1; (**b**) spectral profile of Component 1; (**c**) concentration profile of Component 2; (**d**) spectral profile of Component 2.

For Sample Set 3, Multivariate Curve Resolution–Alternating Least Squares (MCR-ALS) was applied to investigate the compositional changes in camellia oil induced by heating. As shown in Figure 7, the concentration profiles obtained from MCR-ALS decomposition (Figure 7a,c) reveal that the unsaturated fatty acids in camellia oil underwent rapid degradation under high-temperature conditions, while the generation and accumulation of oxidation products accelerated with increasing temperature. Component 1 (presumed to represent oxidation products) exhibited a gradual increase in concentration as the temperature rose, especially becoming more pronounced at higher temperatures, whereas Component 2 (presumed to represent unoxidized fatty acids) showed a significant decrease. This complementary trend indicated a dynamic transition during heating from a system dominated by unoxidized fatty acids to one dominated by oxidation products, reflecting the rapid depletion of unsaturated fatty acids and the accelerated accumulation of secondary oxidation products.

It was observed that when the temperature exceeded approximately 150–180 °C, the Raman signal intensity at 1650 cm^−1^, associated with C=C stretching vibrations, declined sharply, suggesting a substantial increase in the oxidation degradation rate of unsaturated fatty acids. Concurrently, characteristic signals of oxidation products, such as carbonyl groups, were enhanced, further confirming the intensification of oxidative processes under high-temperature conditions. During the initial heating stages, Component 2 predominated; however, with prolonged heating and elevated temperatures, Component 1 gradually became the dominant species.

In addition, the progressive weakening and eventual disappearance of the carotenoid-related Raman peak at 1525 cm^−1^ indicated the thermal degradation of carotenoids, which may contribute to a reduction in the overall antioxidant capacity of camellia oil. Furthermore, thermal oxidation may involve the generation of new compounds or changes in the concentrations of existing constituents, leading to variations in other spectral features and reflecting the differential reactivity and stability of various oil components under thermal stress.

Direct analysis of the spectral data (Figure 7b,d) allowed clear identification of several key characteristic peaks. In the spectral profile of Component 2, the 1650 cm^−1^ peak (cis-C=C stretching vibration) appeared strong, whereas it was markedly reduced in Component 1, indicating a decrease in unsaturated fatty acid content and cis-double bonds with increasing temperature. Similarly, the carotenoid peak at 1525 cm^−1^ progressively disappeared, further elucidating the thermal degradation of carotenoids. Notably, there was a certain degree of spectral overlap between Component 1 and Component 2, reflecting the spectral similarity between degradation products and initial components, a phenomenon commonly observed in complex biological matrices.

In terms of MCR-ALS model quality evaluation, two components were ultimately selected for decomposition modeling. Component 1 (oxidation products) explained 50.7% of the total variance, and Component 2 (unoxidized fatty acids) accounted for 45.6%, yielding a cumulative explained variance of 96.3%. The model’s lack of fit (LOF) was calculated as 3.1%, indicating good model fit and reliability, as summarized in Table 1. Initial spectral estimates were derived from the principal component analysis (PCA) loading matrix, and the final solutions were stable after multiple random initializations, effectively reducing the risk of rotational ambiguity.

In conclusion, the dynamic spectral profiling based on MCR-ALS provided a scientific basis for elucidating the degradation pathways, compositional evolution trends, and key molecular changes of camellia oil under thermal oxidation conditions, offering strong support for the quality control and thermal stability assessment of camellia oil.

#### 2.4.2. Photodegradation Study

**Figure 8:**
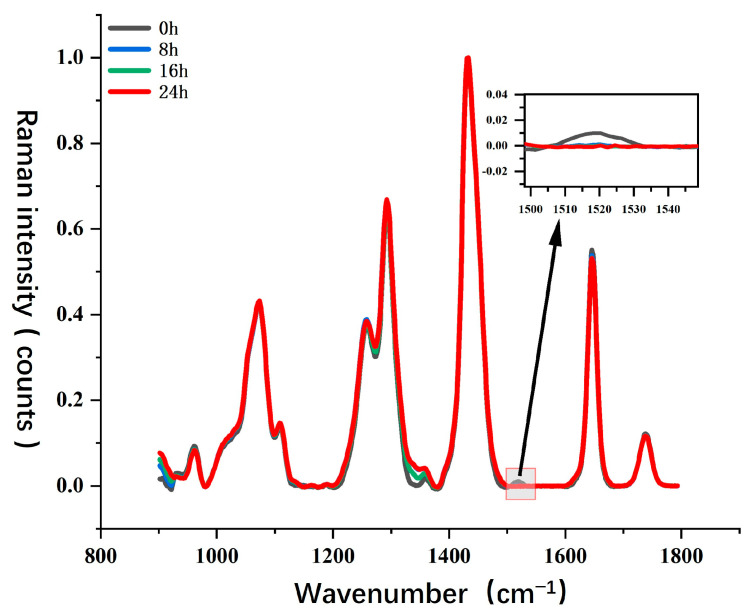
Raman spectra of camellia oil under different UV irradiation times.

For Sample Set 4, this study analyzed the impact of photodegradation on key chemical components of camellia oil, such as carotenoids and unsaturated fatty acids, and utilized Raman spectroscopy to monitor molecular structural changes during the photodegradation process. Figure 8 presents the Raman spectral evolution of camellia oil under different UV exposure durations (0 h, 8 h, 16 h, and 24 h). Overall, the major Raman characteristic peaks of camellia oil remained relatively stable during UV exposure. However, in specific wavenumber regions, photodegradation induced significant spectral changes, particularly at the characteristic peak around 1525 cm^−1^. This peak is typically associated with the C=C conjugated double bond stretching vibration in carotenoids, and its intensity variations can be used to characterize the degradation process of carotenoids.

From the magnified view, it can be observed that the Raman signal at 1525 cm^−1^ is initially relatively strong (0 h). However, as UV exposure time increases, the peak intensity gradually weakens and nearly disappears after 24 h. This phenomenon indicates that UV irradiation significantly accelerates the degradation of carotenoids in camellia oil, likely due to free radical reactions or photooxidation, leading to the breakdown of its molecular structure. Additionally, the variation in the C=C stretching vibration peak of unsaturated fatty acids at 1650 cm^−1^ is relatively minor, suggesting that short-term UV exposure has a limited direct impact on unsaturated fatty acids but may still induce early-stage photooxidation reactions.

UV radiation has high energy, which can induce the photodegradation of key antioxidant components in camellia oil, including unsaturated fatty acids, carotenoids, and tocopherols. Photodegradation primarily involves two mechanisms. First, UV irradiation can trigger photooxidation reactions, where unsaturated fatty acids absorb light energy, generating free radicals and undergoing chain oxidation processes that produce oxidation products such as aldehydes, ketones, and carboxylic acids. This process may accelerate the deterioration of camellia oil quality and affect its sensory properties. Second, UV light can cause the degradation of antioxidants, such as carotenoids and tocopherols, which are natural antioxidants that decompose under light exposure, reducing their ability to scavenge oxidative free radicals. This makes camellia oil more susceptible to oxidative damage and decreases its storage stability. Based on spectral data analysis, UV exposure primarily leads to carotenoid degradation, while the photooxidation process of unsaturated fatty acids is relatively slow. However, with prolonged exposure, oxidation products may gradually accumulate, further accelerating the overall oxidation process of camellia oil.

Compared to thermal oxidation studies, this study found that UV irradiation has a more pronounced effect on carotenoid degradation, whereas the degradation of unsaturated fatty acids is relatively less significant. This suggests that under light exposure, antioxidants in camellia oil degrade preferentially, reducing its resistance to oxidative damage. Additionally, during thermal oxidation, high temperatures can accelerate the auto-oxidation reaction of lipids, resulting in a much higher degradation rate of unsaturated fatty acids compared to photodegradation. Therefore, while UV exposure has a certain impact on camellia oil quality, under high-temperature conditions, the oxidation process is likely to be more severe, further affecting the chemical stability and nutritional composition of camellia oil.

## 3. Materials and Methods

### 3.1. Sample Preparation

To ensure a coherent investigation of authenticity, geographical origin, and quality stability, all samples used in this study were organized into four interrelated sets derived from a common source pool. Camellia oil samples were collected from the Guangxi, Jiangxi, and Hunan provinces—major producing regions in China known for their distinct cultivation environments and representative oil profiles.

(1) Sample Set 1: This set consisted of 30 camellia oil samples (10 from each of Jiangxi Province, Hunan Province, and Gongcheng County in Guangxi Province, China), which were locally collected from small-scale producers. In addition, commercial edible oils used as classification controls—including rapeseed oil, palm oil, corn oil, canola oil, sunflower oil (5 samples of each), and nine olive oil samples—were purchased from JD Supermarket (JD.com Inc., Beijing, China). This set was used for edible oil identification using Raman-based classification models.
(2) Sample Set 2: A subset of 10 camellia oil samples was selected from Set 1, specifically representing the three regions (Guangxi, Jiangxi, and Hunan), and used for geographical traceability analysis. These samples were chosen to examine the correlation between regional origin and compositional Raman spectral features.
(3) Sample Set 3: Camellia oil from Gongcheng, Guangxi (also part of Set 1), was used to evaluate thermal oxidation behavior. In this experiment, 70 mL of camellia oil was heated on an IKA C-MAG HS 7 hot plate with a PT-100 temperature sensor under magnetic stirring (300 RPM). The temperature was gradually raised from 25 °C to 235 °C at a controlled rate of 3 °C/min. A 3 mL aliquot was collected at every 10 °C increment, resulting in 22 samples. Each aliquot was immediately cooled in an ice bath and equilibrated to room temperature before Raman measurement. The heating experiment was performed in triplicate to assess repeatability.
(4) Sample Set 4: To study photodegradation behavior, camellia oil from Gongcheng (also from Set 1) was exposed to continuous ultraviolet (UV) irradiation. A 10 mL sample was placed in a sealed, light-proof chamber and stirred continuously under a UV lamp (wavelength 315–380 nm) positioned 6 cm above the sample. Exposure was maintained at 25 °C for 24 h, and 1 mL aliquots were collected every 8 h for Raman analysis.

This experimental arrangement enabled a unified analytical framework whereby the compositional and spectral characteristics observed in classification and traceability analyses could be directly linked to thermal and photochemical stability behaviors. The detailed composition, geographical sources, and specific applications of each sample set are summarized in Table 2.

### 3.2. Raman Spectroscopy Acquisition

A portable Raman analyzer (Subphotonics Pty Ltd., Zhuhai, China) equipped with a 785 nm, 500 mW laser and a fiber optic Raman probe was used for spectral acquisition. The system operated over a Raman shift range of 200–2000 cm^−1^ with a resolution of 8 cm^−1^. During preliminary testing, the laser power was increased to the maximum output to evaluate potential thermal effects on the samples, and no laser-induced heating or sample degradation was observed. To ensure the reliability of Raman measurements for subsequent chemometric analysis, method validation was conducted by assessing spectral repeatability (RSD < 5% across five replicate measurements per sample), the signal-to-noise ratio, and a comparison with olive oil reference spectra. No fluorescence interference or thermal damage was detected. All spectra were recorded at approximately 25 °C with an integration time of 1 s, and five scans were averaged for each sample to minimize random noise.

Raman spectral preprocessing was conducted using SubRaman 3.0 software. The preprocessing included advanced spike removal to mitigate cosmic ray artifacts generated by the spectrometer. Baseline correction and Savitzky–Golay (S-G) smoothing were applied to enhance the signal-to-noise ratio, addressing baseline drift and high-frequency noise issues.

### 3.3. Data Analysis

This study employed two data analysis methods: Partial Least Squares Discriminant Analysis (PLS-DA) and Multivariate Curve Resolution-Alternating Least Squares (MCR-ALS). These methods were used to classify and differentiate camellia oil from different geographical regions, olive oil, and other edible oils, as well as to analyze the oxidative degradation characteristics of camellia oil under thermal stress.

#### 3.3.1. Partial Least Squares Discriminant Analysis (PLS-DA)

Partial Least Squares Discriminant Analysis (PLS-DA) is a supervised multivariate classification method that extracts feature variables while incorporating sample class information to achieve optimal sample discrimination. Compared with unsupervised methods, PLS-DA maximizes inter-class differences rather than simply focusing on data variance, resulting in higher classification accuracy and interpretability [41,42]. In this study, PLS-DA was applied to the Raman spectral data of various edible oils (including camellia oil, olive oil, and other vegetable oils) to investigate the spectral feature differences among oil samples and evaluate the feasibility of rapid discrimination. To improve data quality and reduce noise interference, all spectral data were subjected to baseline correction and normalization preprocessing prior to modeling, ensuring signal consistency and comparability. Furthermore, to enhance model robustness and discrimination performance, a Variable Importance in Projection (VIP) score-based selection strategy was introduced prior to model construction. Spectral variables with VIP scores greater than 1.0 were selected, allowing the removal of redundant or noisy variables and retaining only those features with significant contributions to classification.

Subsequently, a PLS-DA model was built within the spectral range of 700–1800 cm^−1^ based on the selected characteristic bands, using training set samples for supervised training. The sample distribution among different oil categories was visually analyzed through the resulting score plots. The results demonstrate that the PLS-DA model, based on subtle spectral differences, effectively discriminated camellia oil, olive oil, and other vegetable oils with the aid of the first two latent variables (LVs), providing a reliable analytical tool for the rapid authentication and verification of camellia oil.

#### 3.3.2. Multivariate Curve Resolution-Alternating Least Squares (MCR-ALS)

Under high-temperature conditions, camellia oil undergoes a series of oxidative degradation processes, including changes in fatty acid unsaturation, the formation of peroxides, and the accumulation of secondary oxidation products [43,44]. Due to the overlapping spectral signals of different chemical components during oxidation, a single Raman spectrum alone may not be sufficient to directly analyze the variations in individual components. Therefore, this study employed the MCR-ALS method to analyze the Raman spectral data of camellia oil under different heating durations, extracting the spectral features and concentration trends of key components involved in the oxidation degradation process.

MCR-ALS is a multivariate statistical analysis method based on matrix decomposition, capable of resolving a complex mixed spectral data matrix into the underlying pure component spectra and their corresponding concentration profiles while minimizing the residual error [45,46]. In this study, MCR-ALS was applied to analyze the Raman spectral data of camellia oil during thermal treatment. A non-negativity constraint was imposed during the modeling process to ensure that the extracted concentration profiles and pure component spectra were consistent with chemical reality. The model successfully resolved the key spectral feature patterns of camellia oil at different heating stages. Combined with the dynamic trends observed at critical Raman bands, such as 1650 cm^−1^ and 1525 cm^−1^, the analysis revealed the oxidation and degradation behaviors of unsaturated fatty acids and carotenoid components in camellia oil under thermal stress conditions.

## 4. Conclusions

In this study, portable Raman spectroscopy combined with Partial Least Squares Discriminant Analysis (PLS-DA) and Multivariate Curve Resolution–Alternating Least Squares (MCR-ALS) was used to investigate the authentication, geographical traceability, and stability of camellia oil. The PLS-DA model, based on VIP-selected spectral variables, effectively differentiated camellia oil from other edible oils, with Raman bands at 1250 cm^−1^ and 1650 cm^−1^ contributing significantly to classification. A unique peak at 1525 cm^−1^, identified in samples from Gongcheng, Guangxi, was associated with carotenoids and may serve as a spectral marker for geographical traceability.

Thermal oxidation studies revealed that temperatures above 150 °C caused significant declines in the Raman peaks at 1650 cm^−1^ (unsaturated fatty acids) and 1525 cm^−1^ (carotenoids), indicating molecular degradation. MCR-ALS effectively resolved overlapping spectra and clarified the degradation dynamics under heat. Under UV exposure, the 1525 cm^−1^ peak decreased rapidly and nearly vanished after 24 h, while the 1650 cm^−1^ peak declined during prolonged irradiation, reflecting accelerated oxidative degradation.

Compared to traditional chromatographic techniques, Raman spectroscopy offers rapid, non-destructive, and cost-effective advantages, making it suitable for real-time oil quality control. However, limitations include the relatively small sample set and the use of accelerated experimental conditions. Future work should expand sample diversity, simulate real-world degradation environments, and explore hybrid approaches integrating Raman with NIR, FT-IR, and AI-driven models to enhance prediction accuracy and traceability robustness.

In summary, this study demonstrates that Raman spectroscopy combined with multivariate analysis provides a powerful tool for the rapid evaluation and monitoring of camellia oil quality, with strong implications for food safety, authenticity verification, and market regulation.

## Figures and Tables

**Table 1:** MCR-ALS modeling quality for component changes during thermal treatment of camellia oil.

Component	Chemical Interpretation	Explained Variance (%)	LOF (%)
Component 1	oxidation product component	50.7	-
Component 2	unoxidized fatty acid component	45.6	-
Total		96.3	3.1

**Table 2:** Summary of camellia oil sample sets, regional origins, and analytical applications.

Sample Set	Sample Type	Region(s)	Application	Experimental Details Summary
Set 1	30 camellia oil samples (10 each from Gongcheng, Jiangxi, and Hunan) + 34 control oils (6 types incl. olive)	Guangxi, Jiangxi, Hunan + various	Edible oil identification	Raman spectra collected for classification
Set 2	10 camellia oil samples (selected from Set 1)	Guangxi, Jiangxi, Hunan	Geographical traceability	Focused analysis on regional spectral differences among camellia oil samples
Set 3	Gongcheng camellia oil (from Set 1)	Gongcheng, Guangxi	Thermal oxidation degradation	Heated from 25 °C to 235 °C at 3 °C/min; 3 mL collected per 10 °C; 22 spectra × 3 replicates
Set 4	Gongcheng camellia oil (from Set 1)	Gongcheng, Guangxi	UV photodegradation analysis	UV exposure (315–380 nm) at 25 °C; 1 mL sampled every 8 h over 24 h period

## Data Availability

The original contributions presented in this study are included in the article. Further inquiries can be directed to the corresponding authors.
